# Assessment on Water Quality Parameter and Nutrients Level of Nyatuh River in Relations with *Macrobrachium Rosenbergii* Prawn Populations

**DOI:** 10.21315/tlsr2023.34.1.4

**Published:** 2023-03-31

**Authors:** Nor Azman Kasan, Mhd Ikhwanuddin, Hidayah Manan, Nur Syafirah Zakaria, Amyra Suryatie Kamaruzzan, Ahmad Ideris Abdul Rahim, Ahmad Najmi Ishak

**Affiliations:** 1 Higher Institution Centre of Excellence (HICoE), Institute of Tropical Aquaculture and Fisheries, Universiti Malaysia Terengganu, 21030 Kuala Nerus, Terengganu, Malaysia; 2 STU-Joint Shellfish Research Laboratory, Shantou University, 243 Daxue Road, Shantou, Guangdong, Shantou, China; 3 Faculty of Science and Marine Environment, Universiti Malaysia Terengganu, 21030 Kuala Nerus, Terengganu, Malaysia

**Keywords:** Water parameter, nutrients concentration, Nyatuh River, prawn population, *M. rosenbergii*, Parameter Air, Kepekatan Nutrien, Sungai Nyatuh, Populasi Udang, *M. rosenbergii*

## Abstract

In order to determine the safety level of water parameters and nutrients in the natural environment of fish and freshwater prawn in Nyatuh River, Terengganu, Malaysia, it is necessary to conduct an assessment of water quality parameters. Due to its important, a study was conducted to assess the water quality parameter and nutrients contents from Nyatuh River of Setiu Terengganu in relations to the population of freshwater prawn, *Macrobrachium rosenbergii* caught along the Nyatuh River basin. Total of four expeditions and five stations at different tidal condition for the water quality parameter were assessed during the study. From the results achieved, the overall temperature varied between (26.56°C–29.30°C), dissolved oxygen, DO (3.59 mg/L–6.50 mg/L), pH (4.99–7.01), salinity (0.01ppt–4.22ppt), depth (2.71 m–5.54 m) while for ammonia (0.01 mg/L–0.24 mg/L), nitrite (0.01 mg/L–0.05 mg/L) and phosphate (0.01 mg/L–0.12 mg/L). While the number of prawns caught are 176, 160, 102 and 68 for Expeditions 1, 2, 4 and 3, respectively. Possibly, the heterogeneous number of prawns caught is a result of significant differences in water level depth during high tides and low tides, as well as a fluctuation in the ammonia concentration levels in each of the stations and expeditions. For statistical analysis, the temperature showed no significant difference between the expedition, stations and tidal. That is *p* = 0.280, *p* > 0.05 and F = 1.206, respectively. While dissolved oxygen, DO, showed no significant difference as well, that is *p* = 0.714, *p* > 0.05 and F = 0.737. However, the level of water depth was significantly different between expedition, station and tidal, that is *p* = 0.000, *p* < 0.05 and F = 3.120. Ammonia, on the other hand shows no significant difference between expedition, station and tidal, that is *p* = 0.476, *p* > 0.05 and F = 0.973. The same goes for nitrite and phosphate concentration. There was no significant difference between expedition, stations and tidal, that is *p* = 0.569, *p* > 0.05 and F = 0.879 and *p* = 0.247, *p* > 0.05, F = 1.255, respectively. In Expedition 1, the good water quality parameter and very low ammonia concentration resulted in a larger prawn population as compared to other expeditions. The distribution or mixture of prawns caught is heterogeneous at different stations due to the significant differences in water depth and also to the fluctuation in water quality due to varying ammonia levels. In conclusion, the water quality in Nyatuh River fluctuated across expeditions, stations, and tides, as well as significant differences in water level depths between high and low tides. Due to the rapid growth and importance of industrial and aquaculture operations along the river, extra attention should be devoted to avoid the impact of excessive pollutant in order to protect the ecosystem.

## Abstract (fr)

Penilaian parameter kualiti air perlu dijalankan untuk menentukan tahap keselamatan parameter air dan nutrien dalam persekitaran semula jadi ikan dan udang air tawar di Sungai Nyatuh, Terengganu, Malaysia. Maka, satu kajian telah dijalankan untuk menilai parameter kualiti air dan kandungan nutrien di Sungai Nyatuh, Setiu, Terengganu dalam hubungan dengan populasi udang air tawar, *Macrobrachium rosenbergii* yang ditangkap di sepanjang lembangan Sungai Nyatuh. Sebanyak empat ekspedisi dan lima stesen pada keadaan pasang surut berbeza untuk parameter kualiti air telah dinilai semasa kajian dijalankan. Berdasarkan keputusan yang dicapai, suhu keseluruhan berbeza-beza antara (26.56°C–29.30°C), oksigen terlarut (3.59 mg/L–6.50 mg/L), pH (4.99–7.01), kadar kemasinan (0.01ppt–4.22ppt), kedalaman (2.71 m–5.54 m) sementara bagi ammonia (0.01 mg/L–0.24 mg/L), nitrit (0.01 mg/L–0.05 mg/L) dan fosfat (0.01 mg/L–0.12 mg/L). Manakala bilangan udang yang ditangkap adalah 176, 160, 102 and 68 ekor, masing-masing untuk Ekspedisi 1, 2, 4 dan 3. Bilangan heterogen udang yang ditangkap berkemungkinan adalah hasil daripada perbezaan ketara dalam kedalaman paras air semasa air pasang dan air surut, serta turun naik paras kepekatan ammonia di setiap stesen dan ekspedisi. Bagi analisis statistik, suhu tidak menunjukkan perbezaan yang signifikan antara ekspedisi, stesen dan pasang surut, iaitu *p* = 0.280, *p* > 0.05 dan F = 1.206, masing-masing. Oksigen terlarut juga tidak menunjukkan perbezaan yang signifikan, iaitu *p* = 0.714, *p* > 0.05 dan F = 0.737. Walau bagaimanapun, paras kedalaman air adalah berbeza secara signifikan antara ekspedisi, stesen dan pasang surut, iaitu *p* = 0.000, *p* < 0.05 dan F = 3.120. Ammonia pula tidak menunjukkan perbezaan yang signifikan antara ekspedisi, stesen dan pasang surut, iaitu *p* = 0.476, *p* > 0.05 dan F = 0.973. Begitu juga dengan kepekatan nitrit dan fosfat. Tidak terdapat perbezaan yang signifikan di antara ekspedisi, stesen dan pasang surut, iaitu masing-masing pada *p* = 0.569, *p* > 0.05 dan F = 0.879 dan *p* = 0.247, *p* > 0.05, F = 1.255. Dalam Ekspedisi 1, parameter kualiti air yang baik dan kepekatan ammonia yang sangat rendah menghasilkan populasi udang yang lebih besar berbanding ekspedisi lain. Taburan atau campuran udang yang ditangkap adalah heterogen di stesen yang berbeza disebabkan oleh perbezaan ketara dalam kedalaman air dan juga turun naik kualiti air akibat paras ammonia yang berbeza-beza. Kesimpulannya, kualiti air di Sungai Nyatuh berubah-ubah merentasi ekspedisi, stesen dan pasang surut, serta perbezaan ketara dalam kedalaman paras air antara air pasang tinggi dan rendah. Disebabkan pertumbuhan pesat dan kepentingan operasi perindustrian dan akuakultur di sepanjang sungai, perhatian tambahan harus ditumpukan untuk mengelakkan kesan pencemar yang berlebihan untuk melindungi ekosistem.

**Highlights**
- Water quality monitoring and assessment is important to indicate the quality of pollution in the river as well as an indicator for the prawn population.
- The quality of the water in Nyatuh River fluctuated between expeditions, stations, and tides.
- Heterogeneous mixture for the number of prawns caught caused by the significant differences of water level depth and also by the fluctuation on water quality and ammonia level concentrations.
- Good water quality parameter and very low ammonia concentration in expedition 1 contributed to the higher number of prawn population caught.

## INTRODUCTION

Rivers are vital sources of water for daily human activities such as drinking, agriculture, industrial, and recreational activities (Suratman *et al*. 2015). However, the growth of industrial development, population of humans, extreme land use, aquaculture and agriculture around the world has caused an increase amount of stress on the natural environment (Kitsiou & Karydis 2011). According to the Department of Environment Malaysia, based on their environmental quality assessment in 2013, 473 rivers are polluted (5.3%) while 36.6% are mildly polluted (Zaideen *et al*. 2017). Rapid development yields a lot of waste into the aquatic environment. Moreover, it can even change the balance of the system within its ecology (Alssgeer *et al*. 2018). Degradation of river water caused by organic and inorganic pollution that enters the river system frequently and is eventually transported to the sea (Suratman *et al*. 2016).

Having excellent water quality is necessary for a healthy, clean river and ecosystem. There are some basic conditions that must be met for aquatic life to grow in the water. If conditions are poor, it may cause species populations to die. In addition, if conditions are not optimal, organisms become stressed. Hence, water quality parameters need to be measured in order to discover the quality of river water and therefore it is safe to use for any purpose (Ma’arof & Hua 2015).Many parameters were used to determine water quality. However, in this study, the parameters chosen are *in-situ* parameters which are temperature, dissolved oxygen (DO), pH, salinity and depth. While *ex-situ* parameters are ammonia, nitrite and phosphate which are effective when it comes to accessing water quality (Hairoma *et al*. 2016).

Besides, the relationship between tidal fluctuation and water quality parameters are crucial in order to study the different aspects of ecosystem and natural environment (Kumar *et al*. 2012). The oxygen content of water is affected by temperature. As oxygen levels decrease, the temperature increases. In addition, the river absorbs heat from the tides flowing in from the sea. The inflow of sea water from downstream to upstream causes the water temperature to rise at high tide (Fatema *et al*. 2016). Many organisms become stressed when the water temperature changes abruptly. DO concentration refers to the amount of oxygen present in water (Vasistha & Ganguly 2020) and a crucial indicator of a river health. In most cases, oxygen dissolves quickly in water, however the amount of oxygen dissolved in water varies which is influenced by temperature, atmospheric pressure, and salinity (Zafar *et al*. 2016). Contamination in the water can lower the amount of dissolved oxygen in the water and can affect aquatic life. The percentage of hydrogen ions (H^+^) in a solution is known as pH (Nalado *et al*. 2018). The acidity of water has an impact on the plant and animal life that lives in it. In general, a range of 6.5 to 8.5 is appropriate (Vasistha & Ganguly 2020).

The concentration of dissolved salts in water is referred to as salinity. Aquatic species have evolved to live in specific salinity ranges. The monsoon may cause high precipitation of rainfall which can lower the salinity of water in Peninsular Malaysia (Gasim *et al*. 2015). Ammonia levels that are above the recommended limits can be harmful to aquatic life. Although the ammonia molecule is a necessary nutrient for life, too much of it can build up in the body. This can cause changes in metabolism and an elevation of body pH (Ma’arof & Hua 2015). Nitrite concentration in wastewater are moderate, while in the effluent of nitrifying biological treatment plants can be greater (Uddin *et al*. 2015). Nitrite and phosphate in groundwater can come from both point and non-point sources, such as sewage disposal systems and livestock facilities (Dey *et al*. 2021).

The deterioration in water quality is a huge sign of the river basin’s environmental health worsening. The prevention of pollution in river needs effective monitoring, hence the aim of this study is to evaluate water quality parameters and nutrient content in order to determine the quality trend of Nyatuh River of Setiu Terengganu in relations with the population of giant freshwater prawn, *M. rosenbergii* that might link the water parameter and nutrients level with the prawn populations caught in the expeditions conducted.

## METHODOLOGY

**Figure 1:**
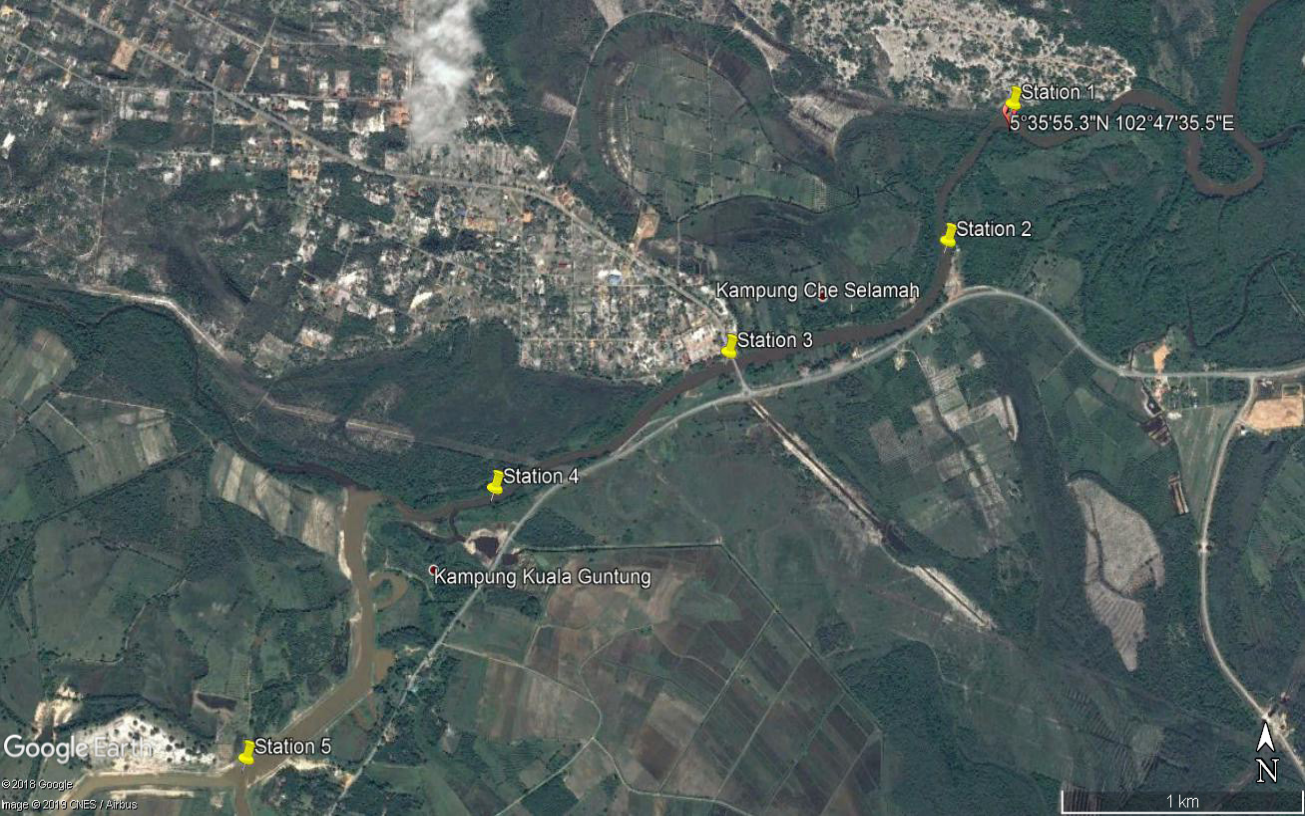
Location of the sampling station at Nyatuh River of Setiu, Terengganu.

There was a total of four expeditions, and each expedition were made at different month and year. For instance, Expedition 1 was conducted in September 2019, while Expedition 2 was conducted in July 2020 followed by Expeditions 3 and 4 which was conducted in November 2020 and April 2021, respectively. The analyses of water quality of Nyatuh River were conducted at all four expeditions. These expeditions were conducted at 5 different stations along the Nyatuh River (Station 1, Station 2, Station 3, Station 4 and Station 5) referring to the specific stations in Fig. 1. The details GPS coordinate is tabulated in Table 1.

Tidal refers to water level and can be differentiated by low tide and high tide using a portable depth meter. The analyses were carried out using the YSI multiprobe Pro Plus for *in-situ* parameters such as temperature, DO, pH, and salinity. For nutrients such as ammonia, nitrite and phosphate, samples were collected triplicates of 1 L water for the water quality analyses. The nutrients concentration was measured using the Shimadzu UV-1800 UV-vis spectrophotometer by applying the phenate method and cadmium reduction method according to the Pearson 1984 protocol (American Public Health Association, APHA 2012).

To assess the giant freshwater prawn population, three methods were used: fishing with a prawn fishing rod, catching with a fishing net, and catching with prawn traps. The same methods for catching prawns were used in every expedition. Sampling was conducted based on low tide and high tide as per the tide chart. For further assessment of the relationship between number of prawns caught and water quality parameters achieved in every expedition, the number of prawns caught and number of prawn samples were recorded in every expedition.

## DATA ANALYSIS

The water quality assessment of all data collected used the Statistical Package for Social Sciences 25.0 (SPSS 25.0) for the analytical analyses. While three-way analysis of variance (ANOVA) was used to determine the interaction effect between three independent variables of expedition, station, and tide on the dependent variables. Such as the temperature, DO, pH, salinity, depth and nutrients (ammonia, nitrite and phosphate).

## RESULTS

**Figure 2:**
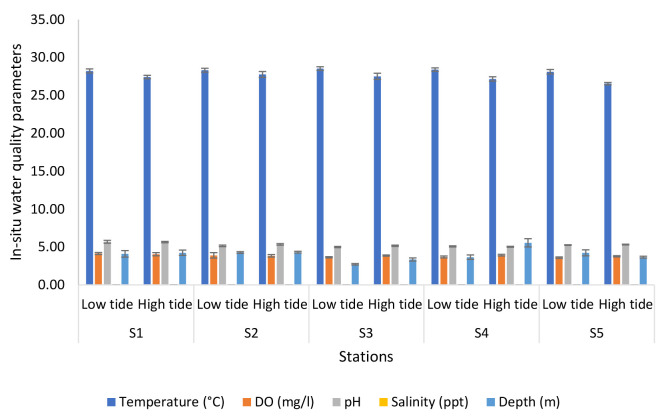
Analyses of *in-situ* water quality parameters of Nyatuh River by different stations and tidal for Expedition 1.

The highest number of prawns caught was during Expedition 1 with 176. This was followed by Expedition 2 with 160 prawns caught, Expedition 4 with 102 prawns and finally, Expedition 3 with 68 prawns. The high prawn catch during Expedition 1 could have been attributed to the good water quality parameter and a very low ammonia concentration. Meanwhile, in Expedition 3 where the lowest number of prawns were caught was due to ammonia concentration (0.10 mg/L–0.39 mg/L) and phosphate concentration (0.04 mg/L–0.15 mg/L) which was slightly high. The significant differences in the water level depth also might contribute to the number of prawns caught during each expedition. For other parameters such as temperature, DO and salinity, the value was not really differentiated between the expeditions. From statistical analysis conducted, the temperature showed no significant difference between the expedition, stations and tidal (*p* = 0.280, *p* > 0.05 and F = 1.206). The same goes for DO, there was no significant difference (*p* = 0.714, *p* > 0.05 and F = 0.737). However, there was a significant difference between expedition, station and tidal (*p* = 0.000, *p* < 0.05 and F = 3.120) in salinity. While ammonia was identified with no significant difference between expedition, station and tidal (*p* = 0.476, *p* > 0.05 and F = 0.973). Nitrite and phosphate concentration has no significant difference as well with *p* = 0.569, *p* > 0.05, F = 0.879 and *p* = 0.247, *p* > 0.05, F = 1.255 between expedition, stations and tidal, respectively. Fig. 2 shows the results of the analyses of *in-situ* water quality parameters of the Nyatuh River at different stations and tides for Expedition 1. The temperature of the low tide increased from Station 1 to Station 3 (28.23°C to 28.54°C) and then decreased at Stations 4 and 5 (28.40°C and 28.12°C). For high tide, the temperature rose at Station 1 and 2 (27.43°C and 27.77°C) and then decreased at Station 3 and Station 5 (27.52°C to 26.56°C). The minimum value of DO of low tide and high tide was at Station 5 which are 3.59 mg/L and 3.76 mg/L. Meanwhile, the maximum value of DO of low tide and high tide at Station 1 are 4.14 mg/L and 4.04 mg/L. The pH values of low tide ranged between 4.99 and 5.68 while for high tide were ranged between 5.02 and 5.64. The salinity of low tide for each station is same which is 0.02 ppt while the minimum value of salinity for high tide was 0.01 at Station 5 and the maximum value was 0.03 at Station 1. Lastly, the depth for low tide and high tide were fluctuated which ranged between 2.71 m and 4.27 m for low tide and 3.34 m and 5.54 m.

**Figure 3:**
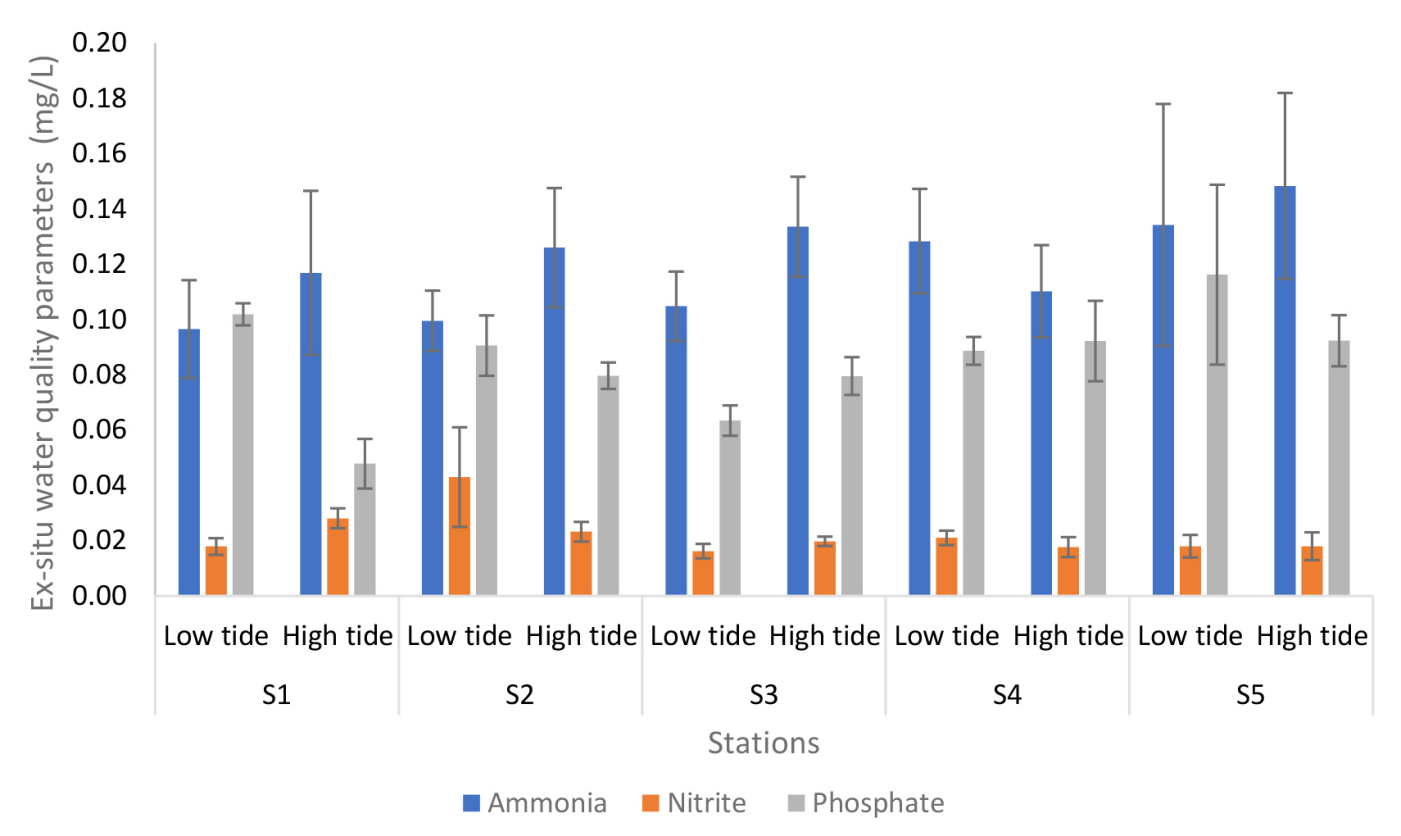
Analyses of *ex-situ* water quality parameters of Nyatuh River at different stations and tidal for Expedition 1. Unit of nutrients level was in mg/L.

Fig. 3 shows the analyses of *ex-situ* water quality parameters of Nyatuh River at different stations and tides. The ammonia during low tide was maintained at 0.10 mg/L and increased at Station 4 and Station 5 which is 0.13. While during high tides, the levels varied between 0.11 and 0.15. On the other hand, the nitrite during low tide has increased at Station 1 and Station 2 (0.02 to 0.04) and remain unchanged at the following stations which is 0.02. While for high tide, the concentration remains the same for each station, which was 0.02 except Station 1 which was 0.03. Lastly, the minimum value of phosphate during low tide was 0.06 at Station 3 and reached a maximum value of 0.12 at Station 5 while during high tide, the minimum value of phosphate was 0.05 and the maximum value was 0.09.

**Figure 4:**
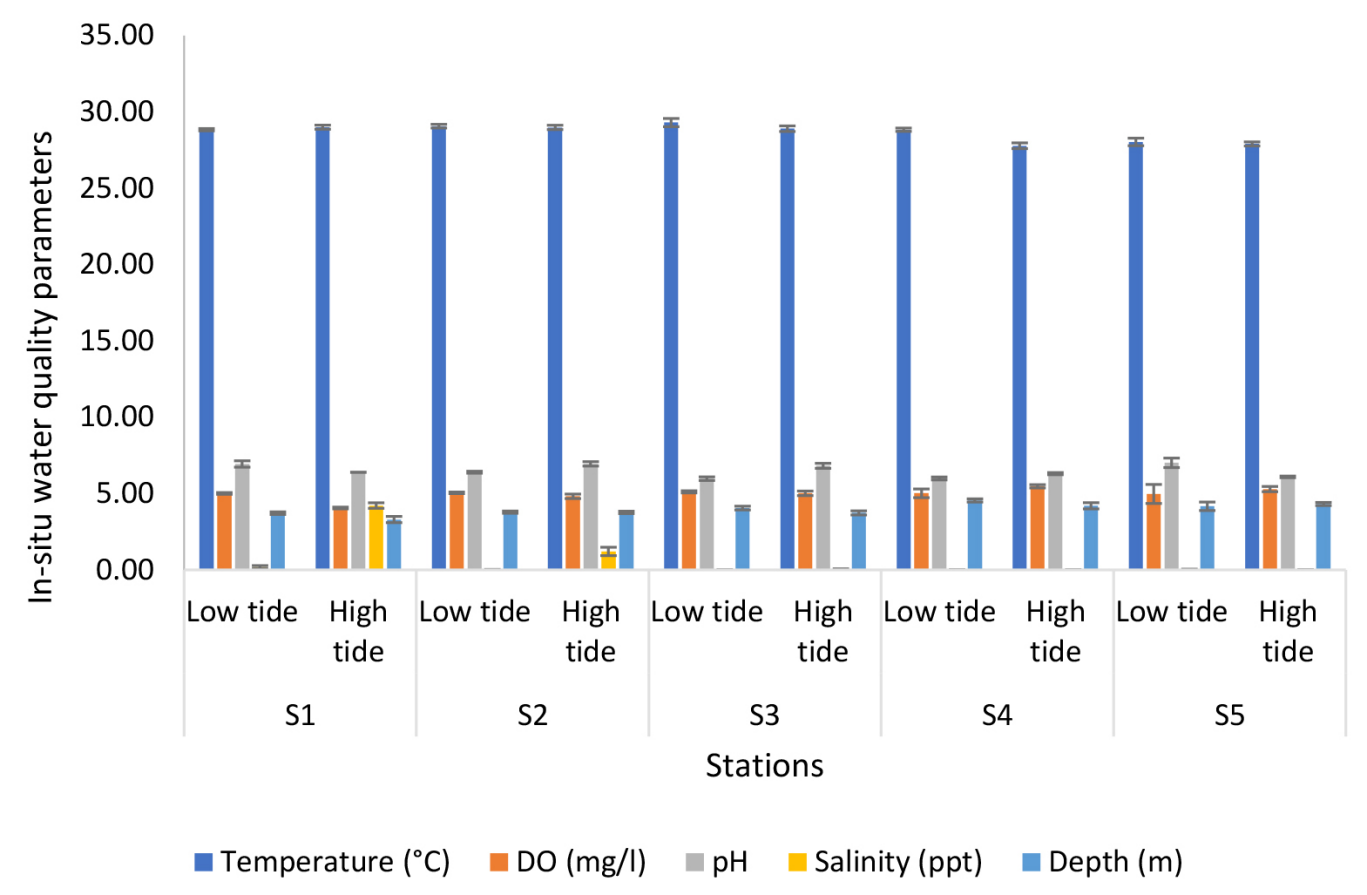
Analyses of *in-situ* water quality parameters of Nyatuh River at different stations and tidal for Expedition 2.

Fig. 4 shows the results of the analyses of *in-situ* water quality parameters of the Nyatuh River at different stations and tides during Expedition 2. The temperature at low tide ranged from 28.83°C to 29.30°C. While at high tide, the temperature ranged from 27.78°C to 29°C. During low tide, dissolved oxygen levels fluctuated (4.98 mg/L to 5.12 mg/L), while levels for high tide increased (4.06 mg/L to 5.30 mg/L).The minimum value of pH for low tide and high tide at Station 4 are 5.99 and 6.31, respectively. While the maximum value for low tide was 7.01 at Station 5 and high tide at Station 2 which is 6.95. The salinity of low tide decreased until Station 4 and then increased at Station 5 which is 0.04 ppt. While the salinity for high tide decreased rapidly from 4.22 ppt at Station 1 to 0.02 ppt at Station 5. Lastly, the depth for low tide ranged between 3.72 m and 4.55 m, for high tide ranged between 3.30 m and 4.32 m.

**Figure 5:**
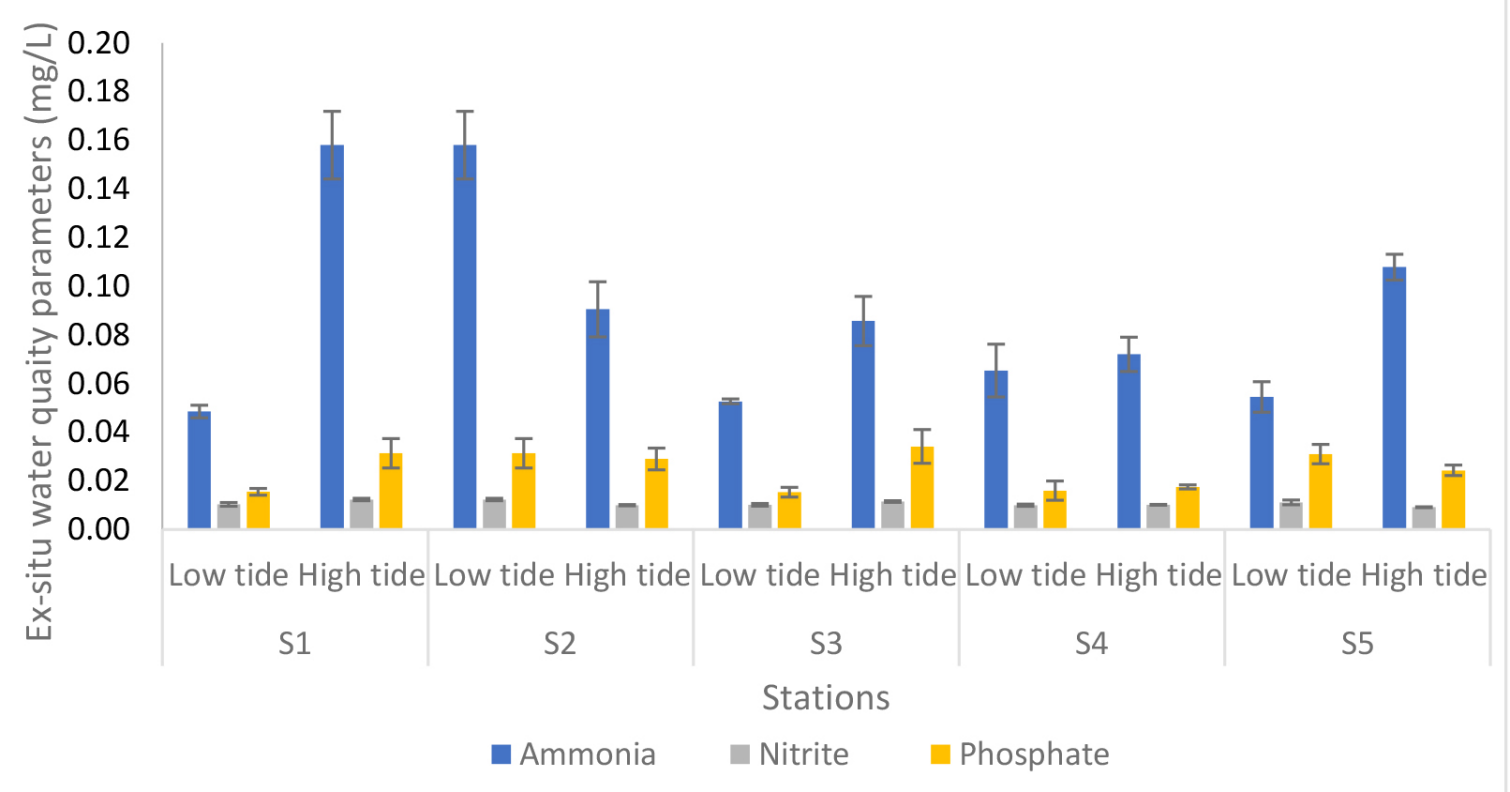
Analyses of *ex-situ* water quality parameters of Nyatuh River at different stations and tidal for Expedition 2. Unit of nutrients was in mg/ L.

Fig. 5 illustrated the analyses of *ex-situ* water quality parameters of Nyatuh River at different stations and tidal. The ammonia content during low tide was maintained at Station 1, Station 2, Station 3 and Station 5 which is 0.05 except at Station 4 which is 0.07. During high tide, the values varied between 0.07 and 0.16. The nitrite content during low tide and high tide for each station remains unchanged which is 0.01. Lastly, the phosphates content during low tide remains the same until Station 4 which was 0.02 and then increased at Station 5 which is 0.03. During high tide, the phosphates were maintained at Station 1 to Station 3 which is 0.03 and decreased at Station 4 and Station 5 which is 0.02.

**Figure 6:**
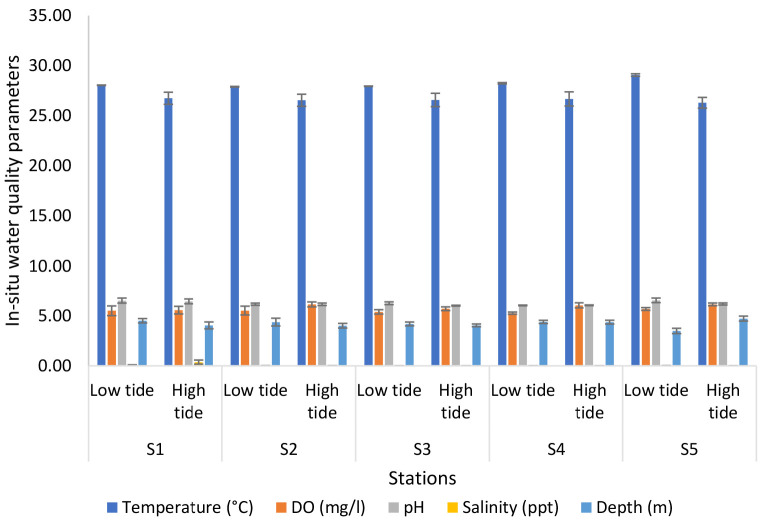
Analyses of *in-situ* water quality parameters of Nyatuh River by different stations and tidal for Expedition 3.

Fig. 6 revealed the result for the analyses of *in-situ* water quality parameters of Nyatuh River by different stations and tidal for Expedition 3. The temperature of each station was quite even, which are between 27.90°C and 29.07°C for low tide while the temperature obtained during high tide lies between 26.30°C and 26.68°C. The minimum value of DO for low tide was 5.26 mg/L at Station 4 and the maximum value was 5.68 mg/L at Station 5. While during high tide, the minimum value of DO was 5.56 mg/L at Station 1 and the maximum value was 6.14 mg/L at Station 5. The pH value for each station during low tide (6.03 to 6.54) and high tide (6.01 to 6.44) were maintained steadily. The salinity of low tide decreased (0.10 ppt to 0.02 ppt) until Station 4 and then increased at Station 5 which is 0.03 ppt while the salinity for high tide decreased from 0.37 ppt at Station 1 to 0.02 ppt and stayed the same through the following stations. Lastly, the depth for low tide ranged between 3.48 m and 4.50 m, for high tide ranged between 4.01 m and 4.71 m.

**Figure 7:**
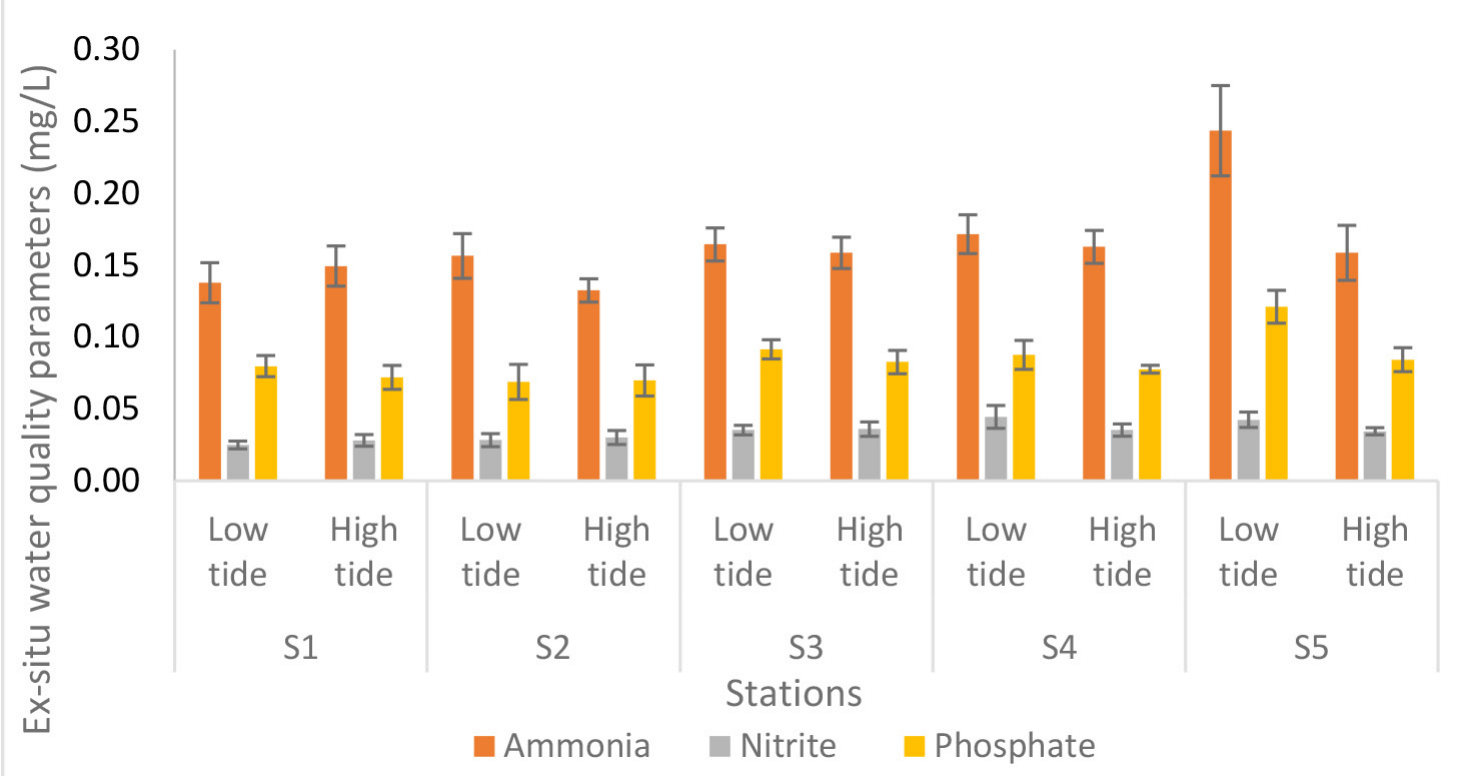
Analyses of *ex-situ* water quality parameters of Nyatuh River at different stations and tidal for Expedition 3. Unit of nutrients was in mg/L.

Fig. 7 illustrate the analyses of *ex-situ* water quality parameters of Nyatuh River at different stations and tidal. The ammonia content during low tide increased from Station 1 to Station 5 which is 0.14 mg/L to 0.24 mg/L while during high tide, the content remains unchanged for each station which is 0.16 except for Stations 1 and 2 which are 0.15 and 0.13, respectively. The nitrite content during low tide and high tide is the same for Stations 1 and 2 which is 0.03. While at Stations 3 and 4, the nitrite content is 0.04. However, during low tide, the nitrite content was 0.04 at Station 5 and 0.03 during high tide. Lastly, the phosphate content values during low tide at each station was varied between 0.07 and 0.12 as well as at high tide which is between 0.07 and 0.08.

**Figure 8:**
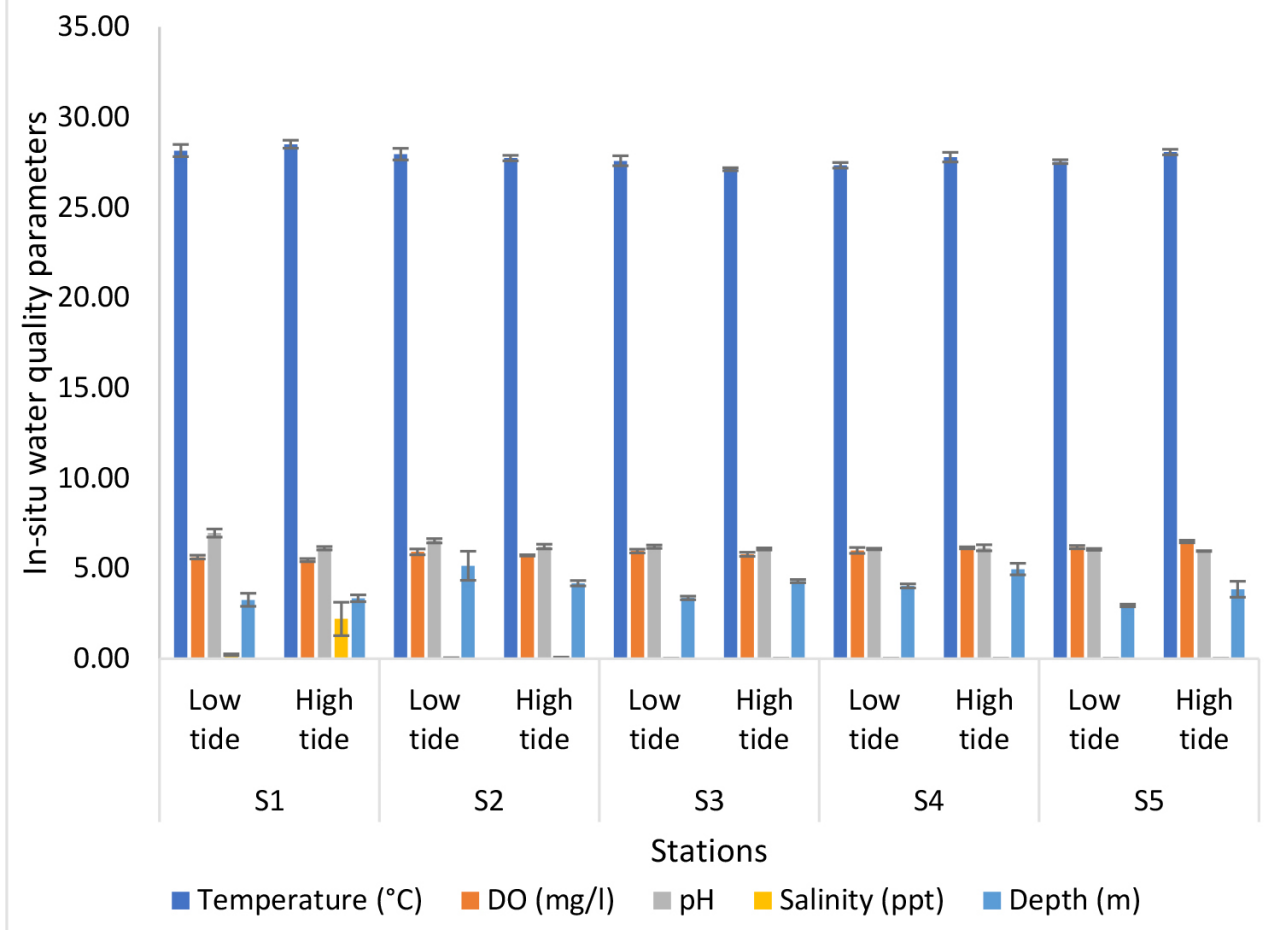
Analyses of *in-situ* water quality parameters of Nyatuh River at different stations and tidal for Expedition 4.

Moreover, Fig. 8 displayed the result for the analyses of *in-situ* water quality parameters of Nyatuh River at different stations and tidal for Expedition 4. The temperature during low tide dropped from 28.15°C at Station 1 to 27.33°C at Station 4. However, the temperature increased at Station 5 which is 27.53°C. During high tide, the temperature varied between 27.12°C and 28.50°C. The values of DO for low tide increased from Station 1 (5.63 mg/L) to Station 5 (6.18 mg/L). The same goes for high tide. The DO value increased from Station 1 (5.46 mg/L) to Station 5 (6.50 mg/L). Furthermore, the pH values dropped during low tide; from 6.96 at Station 1 to 6.06 at Station 5. However, during high tides, the pH values fluctuated between 5.67 and 6.22. The salinity of low tide dropped as well at Station 1 (0.22 ppt) until Station 3 (0.02 ppt/) and stayed the same through the following stations. However, during high tides, the salinity value dropped from 2.21 ppt at Station 1 to 0.02 ppt and remain unchanged until Station 5. Lastly, the depth for low tide ranged between 2.95 m and 5.15 m. While during high tide, the depths ranged between 3.35 m and 4.97 m.

**Figure 9:**
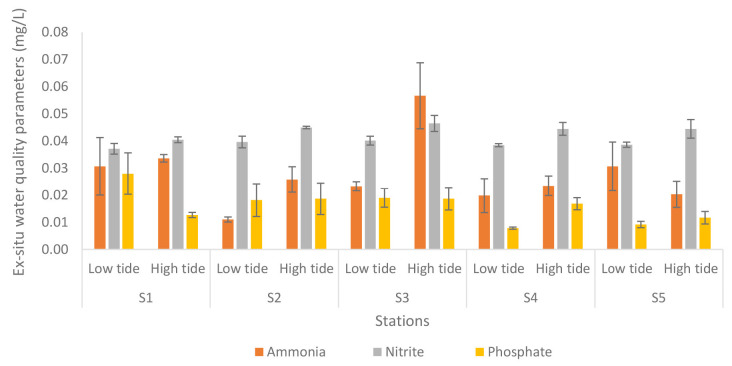
Analyses of *ex-situ* water quality parameters of Nyatuh River by different stations and tidal for Expedition 4. Unit of nutrients was in mg/L.

Fig. 9 shows the results of *ex-situ* water quality tests at different stations and tides of the Nyatuh River. Ammonia levels fluctuate between 0.01 and 0.03 during low tide, but remain unchanged at 0.03 at Stations 1 and 2 during high tide. Stations 4 and 5 have an ammonia content of 0.02, except Station 3 with 0.06. Nitrite levels are consistent at 0.04 for all stations during low and high tide, except for Stations 2 and 3, which have a nitrite level of 0.05 during high tide. Phosphate levels decrease from 0.03 to 0.01 during low tide, and range from 0.01 to 0.02 during high tide.

## DISCUSSION

According to Kurup and Harikrishnan (2020), it is important to monitor the stocking population of the giant freshwater prawn, *M. rosenbergii*, to understand its response to ecological changes in its habitat. The highest prawn population was recorded during Expedition 1. The salinity was in an ideal range for freshwater (0.01–0.04) and pH was also optimal (4.77–6.75). Ammonia levels were low and almost reached zero (0.00 mg/L–0.37 mg/L), which may have contributed to the higher prawn population observed during Expedition 1 compared to other expeditions. The lowest number of prawns caught was during Expedition 3, which was around 68. The nutrients level of ammonia (0.1 mg/L–0.39 mg/L) and phosphate (0.04 mg/L–0.15 mg/L) was a little bit higher during this expedition compared to other expedition that might trigger stress to the prawn that resulted in the lower prawn population caught during Expedition 3. According to Gasim *et al*. (2015) and O’Brien (1995), they found out that some disturbance on the water quality of the freshwater such as by the influx of the saline water might give stress to the fish especially to the juvenile and egg stages that might not be able to tolerate the water quality fluctuation. Thus, the disturbance on the water quality in term of nutrients concentration of a bit higher ammonia and phosphate concentration than other expeditions might be the reason for the lower prawn population caught in Expedition 3.

Temperature is crucial for aquatic life due to its wide range of temperature tolerance. However, temperature may affect DO differently in polluted water, potentially due to chemical and biological processes, as well as man-made structures like dams and weirs (Uddin *et al*. 2015). The results from each expedition show that low tide temperatures are slightly higher than high tide temperatures, fluctuating between 26°C and 29°C. These temperatures are below 32°C and suitable for aquatic life in the river. Temperatures in warm water streams should not exceed 32°C, as high temperatures reduce DO and can lead to fish death (Rauf 2010).

DO is a water quality parameter that measures the amount of oxygen dissolved in water in contact with air in the atmosphere. It is essential for respiration. A level of DO below 1 mg/L is unfavourable for aquatic life and suggests water pollution, while a normal level should be 6 mg/L or higher (JAS 2008). The results of the analysis revealed that DO values for the five stations ranged from 3 mg/L to 6 mg/L, indicating low levels of water pollution. DO values were higher during low tide at Stations 1 to 3 compared to high tide, while the opposite was observed at Stations 4 and 5. These differences may be due to the presence of cooler water and higher water flow, which can hold more DO than slower water (Kasan 2006).

The pH measures the acidity or basicity of minerals and organic matter in rivers. Its range of values is 0 to 14, and it is crucial in determining the viability of organisms and bacteria, as high or low pH levels can be harmful. The National Water Quality Standard (NWQS) for Malaysia considers a normal pH range for water supply and habitats for sensitive aquatic species to be 6.5 to 9.5. This study shows average pH values approaching the normal range, with a minimum of 4.99 at Station 3 of Expedition 1 and a maximum of 7.01 at Station 5 of Expedition 2 (low tide). The low pH value in water is due to high concentrations of ammonium, nitrate, and acid sulphate soils (Xie 2004).

Salinity measures dissolved salts in water. High levels of dissolved matter make water unsuitable for common uses (Uddin *et al*. 2015). Results showed variation in salinity values across stations. The lowest value was 0.02 ppt, while the highest was 4.22 ppt during high tide at Station 1 in Expeditions 2 and 4. This is likely because Station 1 is closer to the sea than other stations, and the faster water flow during high tide carries saltwater from the sea into the station area. Tidal limits of rivers in specific areas that flow into the sea cause fluctuations in salinity between high and low tide. Other factor that contributes may be the speed of water flow during high tide that caused salt water from the sea entering into area of the station.

The analysis of *ex-situ* water quality of nutrient concentration showed fluctuating values for ammonia at each station. The highest value, 0.24 mg/L, was at Station 5 during low tide in Expedition 3. The highest concentration of ammonia was found in the upper zone of the river during low tide, likely due to anthropogenic activity and sewage inputs in the area. Nitrite values were slightly stable at Station 1 until Station 5 which ranged from 0.01 mg/L to 0.05 mg/L, below the 0.25 mg/L limit recommended for prawn culture. Phosphate values varied and were mostly high during high tide at Stations 1 to 5. The range was between 0.01 mg/L and 0.12 mg/L, within the recommended limit of 0.05 mg/L to 0.5 mg/L for shrimp culture. The high phosphate content around 0.12 mg/L during Expedition 3 at Station 5 may have been due to agricultural and livestock activities in the area. However, overall, the nutrient concentrations were within the recommended values and safe for all expeditions.

## CONCLUSION

Water quality monitoring and assessment is important for determining the level of pollution in the Nyatuh River and as an indicator for the prawn population. The highest prawn catch was during Expedition 1, which may be due to favourable water quality parameters and nutrient levels. Conversely, the lowest prawn population during Expedition 3 was caused by significant differences in water depth during high and low tide, as well as slightly higher levels of ammonia and orthophosphate, which may have stressed the prawns and led to a decrease in population. There is heterogenous mixture for numbers of prawn caught between each expedition which non-uniform distribution of number of prawns caught that caused by the significant differences of water depth during high and low tide and also might triggered by the water quality of ammonia fluctuation. Meanwhile, the dial variation of water quality parameters presented a clear tidal signature. This study also revealed that the concentration of several water quality parameters in the river water has affected by the tide. From the above results this study revealed that water quality parameters which are temperature, DO, pH, salinity and nutrient concentrations showed no significant different among expedition, sampling stations and tides except for depth that display a significant different. Although there are increasing and diminishing patterns, water quality levels have remained reasonably steady. The assessment of pollution and pollution control should be proposed in order to reduce the effect of pollution in the Nyatuh River.

## Figures and Tables

**Table 1:** GPS coordination for each sampling station of Station 1 until Station 5 of Nyatuh River, Setiu, Terengganu.

No.	Station	GPS coordinate	Location
1	Station 1	N 05°35.921′ E 102°47.592′	downstream
2	Station 2	N 05°35.628′ E 102°47.439′	upstream
3	Station 3	N 05°35.386′ E 102°46.934′	upstream
4	Station 4	N 05°35.103′ E 102°46.401′	upstream
5	Station 5	N 05°34.532′ E 102°45.844′	upstream

## References

[b1-tlsr-34-1-51] Alssgeer HMA, Gasim MB, Hanafiah MM, Ali Abdulhadi ER, Azid A (2018). GIS-based analysis of water quality deterioration in the Nerus River, Kuala Terengganu Malaysia. Desalination and Water Treatment.

[b2-tlsr-34-1-51] American Public Health Association (APHA) (2012). Standard methods for the examination of water and wastewater.

[b3-tlsr-34-1-51] Dey S, Botta S, Kallam R, Angadala R, Andugala J (2021). Seasonal variation in water quality parameters of Gudlavalleru engineering college pond. Current Research in Green and Sustainable Chemistry.

[b4-tlsr-34-1-51] Fatema K, Omar WMW, Isa MM (2016). Effects of tidal events on the water quality in The Merbok estuary, Kedah, Malaysia. Journal of Environmental Science and Natural Resources.

[b5-tlsr-34-1-51] Gasim MB, Khalid NA, Muhamad H (2015). The influence of tidal activities on water quality of Paka River Terengganu, Malaysia. Malaysian Journal of Analytical Sciences.

[b6-tlsr-34-1-51] Hairoma N, Toriman ME, Barzani MG (2016). Trend of six physiochemical water quality parameters between 2012 and 2015 of the Marang River, Terengganu, Malaysia. Iranica Journal of Energy and Environment.

[b7-tlsr-34-1-51] Jabatan Alam Sekitar (JAS) (2008). Laporan kualiti alam.

[b8-tlsr-34-1-51] Kasan NA (2006). Kualiti air sungai berdasarkan analisis kimia.

[b9-tlsr-34-1-51] Kitsiou D, Karydis M (2011). Coastal marine eutrophication assessment: A review on data analysis. Environment International.

[b10-tlsr-34-1-51] Kumar P, Tsujimura M, Nakano T, Minoru T (2012). The effect of tidal fluctuation on ground water quality in coastal aquifer of Saijo plain, Ehime Prefecture, Japan. Desalination.

[b11-tlsr-34-1-51] Kurup BM, Harikrishnan M (2020). Reviving the *Macrobrachium rosenbergii* (de Man) fishery in Vembanad Lake, India. NAGA, The ICLARM Quarterly.

[b12-tlsr-34-1-51] Ma’arof H, Hua AK (2015). Kualiti air Sungai UTM: Satu tinjauan awal berpandukan enam parameter Indeks Kualiti Air. Geografia –Malaysian Journal of Society and Space.

[b13-tlsr-34-1-51] Nalado AM, Kamarudin MKA, Wahab NA, Rosli MH, Saudi ASM (2018). Assessment of individual water quality index parameter in Terengganu River, Malaysia. Journal of Fundamental and Applied Sciences.

[b14-tlsr-34-1-51] O’Brien T, Banens RJ, Lechane R (1995). Assessment of the impact of salinity od saline drainage on key fish species. Riverine environment research forum.

[b15-tlsr-34-1-51] Rauf HN (2010). Assesment on quantity and quality of stormwater runoff for urban drainage system and detention pond facilities.

[b16-tlsr-34-1-51] Suratman S, Hussein ANAR, Mohd Tahir N, Latif MT, Mostapa R, Weston K (2016). Seasonal and spatial variability of selected surface water quality parameters in Setiu Wetland, Terengganu, Malaysia. Sains Malaysiana.

[b17-tlsr-34-1-51] Suratman S, Mohd Sailan MI, Hee YY, Bedurud EA, Latif MT (2015). A preliminary study of water quality index in Terengganu River Basin, Malaysia. Sains Malaysiana.

[b18-tlsr-34-1-51] Uddin MN, Alam MS, Mobin MN, Miah MA (2015). An assessment of the river water quality parameters: A case of Jamuna River. Journal of Environmental Science and Natural Resources.

[b19-tlsr-34-1-51] Vasistha P, Ganguly R (2020). Water quality assessment of natural lakes and its importance: An overview. Materials Today: Proceedings.

[b20-tlsr-34-1-51] Xie BD (2004). Impact of the intensive shirmp farming on the water quality of the adjacent coastal creeks from Eastern China. Marine Pollution Buletin.

[b21-tlsr-34-1-51] Zafar MA, Haque MM, Aziz MSB, Alam MM (2016). Study on water and soil quality parameters of shrimp and prawn farming in the southwest region of Bangladesh. Journal of the Bangladesh Agricultural University.

[b22-tlsr-34-1-51] Zaideen MIM, Suratman S, Mohd Tahir N (2017). The evaluation of spatial variation of water quality in Setiu River basin, Terengganu. Sains Malaysiana.

